# Vitamin D, the autonomic nervous system, and cardiovascular risk

**DOI:** 10.14814/phy2.12349

**Published:** 2015-04-22

**Authors:** Michelle Catherine Mann, Morley D Hollenberg, David A Hanley, Sofia B Ahmed

**Affiliations:** Cumming School of Medicine, University of CalgaryCalgary, Alberta, Canada

Cardiovascular disease (CVD) and vitamin D deficiency are extremely prevalent worldwide (Holick 2007; World Health Organization 2011). The potential link between vitamin D deficiency and CVD-related death in both healthy and diseased populations is a growing area of translational research. Historically, the traditional role of vitamin D in maintaining calcium homeostasis and mineral metabolism has been extensively studied (Lieben and Carmeliet 2013). Vitamin D, via its classical genomic transcriptional activity, is known to affect the physiological function of a number of target organs such as the heart, bone, and kidneys (Lieben et al. 2011). Furthermore, as highlighted in a paper published recently in Physiological Resports (Foong et al. 2014), there appears to be a link between vitamin D deficiency and respiratory symptoms in chronic lung disease. In addition, a number of nongenomic, rapid action roles of vitamin D are now known to affect tissue function, which fall outside the traditional role of regulating mineral metabolism (Gniadecki 1998; Brown et al. 1999; Norman et al. 2002; Haussler et al. 2011). While calcium and phosphate have long been regarded as the major players in the interactions observed between mineral metabolism dysfunction and CVD-related deaths, (Peacock 2010; Bolland et al. 2011; Brini et al. 2013), these newer nongenomic roles outside of the traditional target organs suggest that vitamin D itself may have intrinsic actions outside the realm of mineral metabolism (Gniadecki 1998; Brown et al. 1999; Norman et al. 2002; Haussler et al. 2011).

## Vitamin D Metabolism

Vitamin D encompasses a group of fat-soluble prohormones which can be obtained by the body through both the skin via sunlight exposure and through intestinal absorption of dietary sources and supplements (Holick 2007). Although each source contributes to the overall maintenance of vitamin D levels, exposure to sunlight is the greatest resource for the synthesis of this prohormone in humans. Skin exposed to solar ultraviolet B (UVB, 290–320 nm) rays is stimulated to convert 7-dehydrocholesterol into the vitamin D precursor, previtamin D_3_ (Norman et al. 2002). Nutritional animal and fungal sources of vitamin D (cholecalciferol, vitamin D_3_; and ergocalciferol, vitamin D_2_, respectively) undergo two enzymatic hydroxylations within the vitamin D metabolic pathway: (1) in the liver to 25-hydroxyvitamin D; (2) in the kidney, and to a much lesser degree in nonrenal tissues), the enzyme 1*α*-hydroxylase converts 25-hydroxy vitamin D to the biologically active form of vitamin D, 1,25-dihydroxy vitamin D (calcitriol) (DeLuca 2004).

Aside from these naturally occurring vitamin D metabolites, numerous other activated vitamin D formulations have been synthesized and are commercially available (Melamed and Thadhani 2012). For example, alfacalcidiol (1*α*-hydroxyvitamin D_3_) is easily integrated into the regular vitamin D metabolic pathway and is commonly substituted for calcitriol in clinical practice (Melamed and Thadhani 2012). Though observational studies have reported differences in survival associated with various analogs of calcitriol (Teng et al. 2003), no specific vitamin D supplement has been definitively proved to be superior in decreasing risk of all-cause and CVD-related mortality. Classically, vitamin D is known to tightly regulate the release and maintenance of calcium stores and extracellular fluid calcium ion concentration. It does so by stimulating intestinal absorption of calcium and releasing calcium from the skeletal tissue by regulating osteoclast differentiation and bone turnover (Holick 2007; Lieben et al. 2011).

As the primary regulator of calcium, the parathyroid gland plays a major role in vitamin D metabolism. If low circulating levels of calcium are detected by the chief cells of the parathyroid gland, parathyroid hormone (PTH) is released and stimulates the enzymatic production of 1,25-dihydroxy vitamin D within the proximal tubule of the kidney, where the highest concentration of 1-*α* hydroxylase exists within the body (Holick 2007; Lieben et al. 2011). The spike in circulating 1,25-dihydroxy vitamin D causes a release of calcium and phosphate from the bone as well as an increase in absorption of these minerals from the small intestine, which feed back to the parathyroid until the Chief cells detect increased levels of serum calcium. Under normal conditions, levels of vitamin D within the recommended target range (20–60 ng/mL 25-hydroxy vitamin D) will maintain normal serum calcium levels and the chemoreceptors on the Chief cells will not be overly stimulated (DeLuca 2004). However, in the presence of vitamin D deficiency (25-hydroxy vitamin D ≤ 50 nmol/L or ≤20 ng/mL), (Holick et al. 2011) this physiological axis is disrupted.

## Vitamin D Metabolism in Chronic Kidney Disease

In the setting of chronic kidney disease (CKD), as nephron mass declines so does the functional capacity of the renal tissue to regulate the production of active vitamin D metabolites. Quite often, disordered mineral metabolism develops (Kim et al. 2014) and the prevalence of severe vitamin D deficiency is extremely high within the CKD population (Holick 2007; Kim et al. 2014). The coexisting prevalence of vitamin D deficiency, reduced production of calcitriol, and CVD-related mortality in CKD provides an excellent clinical setting to observe and study the potential physiological mechanisms behind the interaction between vitamin D metabolism and CVD-related mortality that is observed in various patient populations globally (Drechsler et al. 2010; Kramer et al. 2012; Wang et al. 2012a; Schottker et al. 2013; Kim et al. 2014).

## Vitamin D and the Autonomic Nervous System

Aside from its traditional roles, vitamin D has also been shown to mediate a number of additional nongenomic or “rapid” actions at the nontranscriptional level in a variety of tissues (Wang et al. 2012b). These effects involve transmembrane receptors and associated secondary messenger systems (G-proteins) (Severson and Hollenberg 1997) that activate intracellular pathways and cascades specific to transmission of action potential and muscular contractions, including calcium regulation via ligand-gated (IP_3_) calcium channels and intracellular vesicular calcium stores (Boland 2011; Buitrago et al. 2013). Interestingly, activated 1,25-dihyroxy vitamin D has been shown to coordinate additional molecular mechanisms, including inflammatory signaling and biosynthesis of neurotransmitters, in areas of the central nervous system which regulate cardiovascular activity (Garcion et al. 2002; DeLuca et al. 2013). A study by Santillan et al. (1999) also demonstrated that *β*-adrenergic signal transduction in chick myocardial cells is enhanced when the cells are placed in a 1,25-dihydroxyvitamin D_3_ bath, suggesting that basic function of the cardiovascular system may rely in part on vitamin D to facilitate arrhythmic signals not only within the higher central nervous system but also at the level of the heart.

In mammals, the autonomic nervous system (ANS) is a subset of the peripheral nervous system, a section of the central nervous system housed within the spinal cord and brain (Barron and Mesh 1996; Sleight 1997). The ANS controls involuntary processes throughout the body, including digestion, respiration, and most relevant to this review, cardiovascular function. Two specific branches of the ANS contribute to the maintenance of cardiovascular function: (1) The cardiac sympathetic plexus and (2) The vagal (parasympathetic) nerve. Both of these systems innervate the heart at the sino-antrial node neuromuscular junction and work in concert with one another to release neurotransmitters which increase or decrease, respectively, the strength and speed of the contractility of the heart (Barron and Mesh 1996; Sleight 1997). In general, changes in the duration of time between each heartbeat can be attributed to shifts in balance between ANS stimulatory and inhibitory activity. This balance and contribution from either limb of the cardiac ANS can then be quantified with sophisticated computer software (Task Force of the European Society of Cardiology and the North American Society of Pacing and Electrophysiology 1996).

The cardiac ANS in humans is influenced by sex, (Mann et al. 2012) age, (Dekker et al. 1997; Surawicz and Parikh 2002) physical activity, (Soares-Miranda et al. 2014) as well as the presence of comorbidities including chronic kidney disease (Ranpuria et al. 2008; Katsilambros 2010; Chan Oct 2008). However, even in healthy populations cardiac ANS dysfunction has independently been shown to be a predictor of sudden cardiac death (Tsuji et al. 1994; Thayer et al. 2010). Specifically, it has been shown in the CKD population that an increase in sympathetic activity accompanied by a significant withdrawal of cardioprotective vagal activity is indicative of significantly increased CVD risk, particularly the risk of sudden arrhythmic cardiac death (Ranpuria et al. 2008; Chan Oct 2008). Interestingly, studies have shown that calcitriol can permeate the blood brain barrier and bind to vitamin D receptors located within the central nervous system (Garcion et al. 2002; Norman et al. 2002; DeLuca et al. 2013). Specifically, the mid-brain and brainstem in which a number of the neurons for the ANS are located have been shown to have an extremely high concentration of vitamin D receptors, thus leading to the hypothesis that vitamin D is actively taken up in these higher central control centers because it plays a specific, noncalcium related role in regulating the ANS (Garcion et al. 2002; DeLuca et al. 2013). These findings, combined with the previously reviewed effect of vitamin D in orchestrating biosynthesis of known neurotransmitters and nervous system activity, suggests that vitamin D may directly contribute to the function of the cardiac ANS, and therefore subsequent CVD-risk, by orchestrating regulatory activity at these higher brain centers of the nervous system.

## Vitamin D Deficiency and Cardiovascular Risk: a Role for the ANS

**Figure 1:**
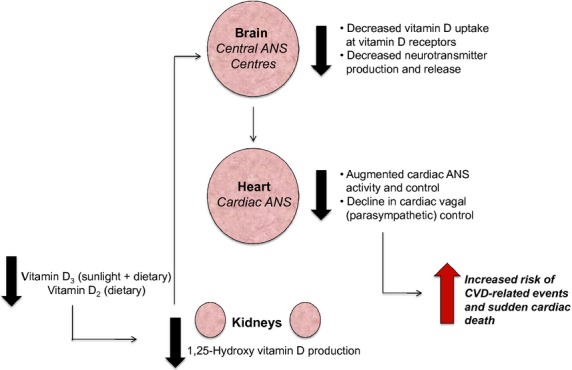
Hypothesized implication of vitamin D deficiency on cardiovascular risk via interaction with the autonomic nervous system.

Vitamin D deficiency is typically defined as a 25-hydroxyvitamin D serum level below 20 ng/mL (50 nmol/L) in the general population, (Holick 2007; Holick et al. 2011) although much controversy exists surrounding the appropriate and optimal vitamin D levels (Institute of Medicine 2011; Pramyothin and Holick 2012; Kidney Disease Improving Global Outcomes 2013). A number of observational studies and meta-analyses of these studies have demonstrated striking associations between vitamin D deficiency and patient-level outcomes including as CVD and CVD-related death, namely sudden arrhythmic death, in both healthy populations and in the high-risk CKD population (Drechsler et al. 2010; Scragg et al. 2010; Kandula et al. 2011; Thadhani et al. 2012; Wang et al. 2012a, 2014; Schottker et al. 2013). Utilizing National Health and Nutrition Examination Survey (NHANES) data from 1988–94 and 2001–06, Scragg et al. (2010) reported that in a cohort of 27,153 individuals with 25-hydroxyvitamin D levels <10 ng/mL demonstrated significantly higher values of mean heart rate, systolic blood pressure, and rate-pressure product, a measure of cardiac work, compared to those with 25-hydroxyvitamin D levels equal to or greater than 35 ng/mL. Unfortunately, very few randomized clinical trials have been prospectively designed to determine causality of vitamin D in regard to important clinical outcomes in any population. The PRIMO trial evaluated the impact of 48 weeks of oral paricalcitol, an activate analog of calcitriol, on the left ventricular mass (LVM) and a number of additional CVD risk factors in a stable chronic kidney disease population (Thadhani et al. 2012). Although no significant relationship between vitamin D therapy and reduction in LVM or other secondary outcomes was observed, the authors reported that a significantly greater number of subjects from the placebo group were admitted to hospital for CVD-related events (Thadhani et al. 2012). The OPERA trial by Wang et al. (2014) reported that 52 weeks of 1ug paricalcitol daily for 52 weeks did not alter LVM in chronic kidney disease patients, however, it did report a reduction in the number of cardiovascular-related hospitalizations compared with placebo. We have also demonstrated in healthy humans that increasing vitamin D levels with 4 weeks of high-dose vitamin D_3_ supplementation (10,000 IU cholecalciferol) was associated with a significant improvement in cardiac ANS balance, specifically enhancing cardioprotective vagal activity, in response to an external stressor (Mann et al. 2014a). Specific to the CKD population, Drechsler et al. (2010) have shown that survival is significantly decreased in end-stage kidney disease patients on hemodialysis with vitamin D deficiency. Those within the lowest quartile of 25-hydroxy vitamin D serum levels had a threefold higher risk of sudden cardiac death compared to those in the highest quartile. Similar results have been reported in a recent study of vitamin D deficient end-stage kidney disease patients on hemodialysis. Krause et al. demonstrated that exposure to short-term artificial sunlight heliotherapy not only increased serum 25-hydroxy vitamin D levels and control of mineral metabolism, but significantly increased measurements of cardioprotective vagal activity which were severely depressed at baseline (Krause 2013). Of note, the magnitude of increase in serum vitamin D levels following heliotherapy was directly correlated to the magnitude of increased cardiac vagal activity, implying that vitamin D levels may indeed constitute an important aspect of the development CVD-related events by interacting directly with the ANS. Further, vitamin D deficiency has been demonstrated to be an important regulator in the neurocardiovascular disease pathways associated with epilepsy (Scorza et al. 2010) and spinal cord injury (Zebracki et al. 2013) in humans. Taken together, these data suggest that vitamin D deficiency directly influences CVD and CVD-related mortality risk by mediating activity of the ANS outside the realm of generic mineral metabolism (Fig.1) in both CKD and non-CKD patient populations. Overall, vitamin D supplementation appears to possess genomic and nongenomic mechanisms that may influence activity of the ANS, presenting an attractive therapeutic option.

## Implications and Future Research

The need for more comprehensive, prospectively designed clinical trials assessing the impact of vitamin D on overall health is highlighted by the current uncertainty in available guidelines which report conflicting data regarding optimal serum vitamin D levels and benefits of achieving such levels with supplementation. Furthermore, a better understanding of the nongenomic roles of vitamin D at the neurological level in relation to the ANS’ known association with CVD and CVD-related death is warranted. The VITAH study (Mann et al. 2014b) (clinicaltrials.gov identifier: NCT01774812) is currently underway and will assess the impact of the activate vitamin D analog alfacalcidiol, with or without additional ergocalciferol (vitamin D_2_) on cardiac ANS activity in a stable end-stage kidney disease population on hemodialysis to determine the effect of the different vitamin D supplementation regimens on cardiac ANS activity. Vitamin D supplementation appears to be a simple and cost-effective treatment to reduce CVD-risk associated by improving the function of the ANS in both healthy and chronic disease populations worldwide. Thus, further translational patient-oriented research is warranted to optimize clinical recommendations and use of vitamin D supplements in high-risk and healthy populations alike.
